# Impact of Virtual Reality–Based Therapies on Cognition and Depression in Patients With Parkinson Disease: Systematic Review and Meta-Analysis of Randomized Controlled Trials

**DOI:** 10.2196/77875

**Published:** 2026-06-30

**Authors:** Yun Zhang, XueLei Li, GuoLi Zhang, HaiYin Zhang, YuXin Xia, XueJie Xu, Ting Sun

**Affiliations:** 1 School of Nursing, Bengbu Medical University, Longzi District, Bengbu, 233000, China, 86 18005529759; 2 Gastroenterology Department, Nantong Third Hospital Affiliated to Nantong University: The Third People's Hospital of Nantong, Nantong, China; 3 Joint Research Center for Regional Diseases of IHM, Bengbu Medical University, Bengbu, China

**Keywords:** virtual reality, Parkinson disease, cognition, depression, quality of life

## Abstract

**Background:**

As a neurodegenerative disorder, Parkinson disease (PD) demonstrates significant prevalence worldwide. As the population ages, the number of patients with PD increases. Individuals with PD are susceptible to varying degrees of cognitive and psychological impairments. Virtual reality (VR)–based therapy is an emerging technology used for cognitive recovery and mental health treatment, yet controversy remains.

**Objective:**

This study aimed to assess the impact of VR-based therapies on cognitive function and depression in patients with PD.

**Methods:**

An extensive database search was conducted through PubMed, Web of Science, Embase, and the Cochrane Library to identify randomized controlled trials (RCTs) that investigated the impact of VR on patients with PD. Studies published before March 31, 2026, which met our inclusion and exclusion criteria, were included. A total of 13 RCTs involving 430 patients with PD were included. The Cochrane risk-of-bias tool was used to assess the risk of bias, indicating the included studies generally had a low risk of bias in randomization but a high or unclear risk concerning allocation concealment and blinding. Random-effects meta-analyses were performed using standardized mean differences (SMDs) with 95% CIs. Hartung-Knapp adjustments were applied, and prediction intervals (PIs) were calculated to assess the expected distribution of effects across future settings. The certainty of evidence was assessed using GRADE (Grading of Recommendations, Assessment, Development, and Evaluation).

**Results:**

In the meta-analysis, VR-based therapies were associated with statistically significant average improvements in global cognitive function (SMD=0.40, 95% CI 0.11-0.70; 95% PI 0.10-0.70; *P*=.01; *I*^2^=0%) and depressive symptoms (SMD=−0.77, 95% CI −1.42 to −0.12; 95% PI −1.82 to 0.27; *P*=.03; *I*^2^=31%). However, the PI for depression crossed the line of no effect, suggesting that this effect may vary across future settings. No significant average effects were observed for executive function (SMD=0.06, 95% CI −0.31 to 0.44; *P*=.66), memory (SMD=0.48, 95% CI −0.30 to 1.25; *P*=.15), attention (SMD=0.01, 95% CI −0.28 to 0.31; *P*=.94), or quality of life (QoL) outcomes (SMD=0.01, 95% CI −0.46 to 0.47; *P*=.97).

**Conclusions:**

The results suggest that VR-based therapies may be associated with improvements in global cognitive function and depressive symptoms in patients with PD, although evidence for executive function, attention, memory, and QoL remains inconclusive. This review provides an updated synthesis that differs from previous reviews by focusing on both global and domain-specific cognitive outcomes, as well as depressive symptoms and QoL, rather than mainly on motor outcomes. By incorporating recent RCTs and considering PIs, risk of bias, and GRADE certainty, this review offers a more cautious interpretation of the evidence. In practice, VR-based therapies may serve as an engaging adjunct to conventional rehabilitation, but larger and methodologically rigorous trials are needed before clinical recommendations can be made.

## Introduction

Parkinson disease (PD) is the second most frequently occurring neurodegenerative condition [1]. In 2019, there were approximately 8.51 million patients with PD worldwide and about 2.84 million in China, making it the country with the largest number of patients. With the progression of China’s aging population, it is estimated that the number of patients with PD in China will reach 4.9 million by 2030, accounting for 57% of the world’s patients with PD [2]. PD manifests through both motor symptoms (resting tremor, slowed movements, rigidity) and nonmotor symptoms (cognitive decline, depression, sleep disorders) [3]. Approximately 60% to 80% of patients with PD develop cognitive impairment. Cognitive decline is a hallmark of the disease and typically manifests prior to the emergence of motor symptoms [4]. Within 3 to 5 years postdiagnosis, mild cognitive impairment emerges in 20% to 57% of individuals with PD, and with the progression of the disease, dementia may occur [5]. A recent study further highlights that the progression from PD-mild cognitive impairment to PD-dementia is a critical transition point, significantly accelerating functional decline and caregiver burden [6]. Once a patient with PD develops dementia, it seriously affects their quality of life (QoL) and imposes a significant caregiver burden on family members. As the disease progresses, cognitive function impairment becomes more severe, and mental health status deteriorates [7]. Mental health includes emotional equilibrium and adaptive functioning, characterized by life satisfaction and effective stress management [8]. Emotional difficulties such as depression, anxiety, and high stress levels are widespread and long-lasting among individuals with PD, and about 40% of them experience depression, which adversely affects rehabilitation and indirectly influences the QoL of patients [9]. Notably, the interplay between cognitive impairment and neuropsychiatric symptoms such as depression is increasingly recognized as a key determinant of overall disease burden and QoL in PD, underscoring the need for integrated management strategies [10].

Virtual reality (VR) is a technology that uses computers to create lifelike simulated environments. Users can actively explore and respond to different situations in a computer-generated 3D world. Difficult real-world conditions can be realistically simulated and experienced through VR platforms [11]. The triad of immersion, interactivity, and imaginative simulation has accelerated the adoption of VR therapeutics in PD management over the past decade [12]. Although several randomized controlled trials (RCTs) support the use of VR therapy to enhance cognitive and mental health outcomes in patients with PD [13-16], opposing studies argue that it offers no additional benefits for cognition or depression compared to traditional rehabilitation [17-19].

Although several systematic reviews have investigated the effects of VR-based therapy on cognitive function in patients with PD, no consistent conclusions have been reached. Dockx et al [12] conducted a meta-analysis of RCTs published before November 26, 2016, and reported potential beneficial effects of VR-based rehabilitation on cognitive outcomes. However, the analysis included only 2 trials assessing cognition, which limited the robustness and generalizability of the findings. Another systematic review [20] examined RCTs conducted prior to December 30, 2018, assessing the efficacy of VR-based therapy on cognitive function in patients with PD. These findings demonstrated that VR intervention showed no superior benefits compared with conventional rehabilitation in cognition. In addition, the psychological benefits of VR have received much attention. A systematic review [21] showed the potential value of VR intervention in reducing depressive symptoms, but due to insufficient data, the findings were described narratively rather than quantitatively synthesized. A meta-analysis by Chuang et al [22] indicated that VR did not improve the depressive state of patients compared with conventional rehabilitation.

In conclusion, previous reviews have generally not provided a comprehensive synthesis of both global and domain-specific cognitive outcomes together with depressive symptoms and QoL [12,21,23,24]. Important cognitive domains, including executive function, attention, and memory, remain insufficiently examined. Since the publication of earlier reviews, several additional RCTs have become available, making an updated synthesis necessary. Furthermore, few previous reviews have interpreted the findings using both CIs and prediction intervals (PIs). Therefore, an updated and targeted systematic review is needed to clarify the average effects of VR-based therapies on cognition and depression, evaluate the expected variability of effects across clinical settings, and assess the certainty and clinical reliability of the current evidence. This review aimed to provide a targeted and updated evaluation of VR-based therapies on global and domain-specific cognitive outcomes, depressive symptoms, and QoL in patients with PD. This study hypothesizes that VR-based therapy holds promise for positively impacting cognition, depression, and QoL in patients with PD.

## Methods

### Protocol

This systematic review was officially recorded in PROSPERO (CRD420251000817). This systematic review was conducted in accordance with the PRISMA (Preferred Reporting Items for Systematic Reviews and Meta-Analyses) guidelines (Checklist 1) [25]. The title of the study was modified after registration. It was revised to *Impact of Virtual Reality–Based Therapies on Cognition and Depression in Patients with Parkinson Disease: Systematic Review and Meta-Analysis of Randomized Controlled Trials* to better reflect the scope and focus of the systematic review.

### Literature Search Strategy

We systematically searched RCTs across 4 major databases (PubMed, Cochrane Library, Web of Science, and Embase). Included studies were restricted to English-language publications (up to March 31, 2026) evaluating VR therapy on cognitive and mental health in patients with PD. The search strategy was developed around 4 key concepts: Parkinson disease, virtual reality–based therapy, cognitive function or depression-related outcomes, and randomized controlled trials. Both controlled vocabulary terms, including MeSH terms in PubMed and Emtree terms in Embase, and free-text terms were used. Field tags and database-specific syntax were adapted for each database. All retrieved records were exported to EndNote 21 (Clarivate) for deduplication. Duplicates were removed using the software’s duplicate identification function and then checked manually. The search strategies are detailed in Multimedia Appendix 1.

### Inclusion Criteria

#### Types of Trials

Our inclusion criteria were limited to peer-reviewed RCTs published in English.

#### Types of Participants

The research subjects were those diagnosed with idiopathic PD according to established clinical diagnostic criteria, such as the UK Parkinson’s Disease Society Brain Bank Clinical Diagnostic Criteria. No limitations were placed on PD population specifics to capture the maximum number of meta-analyses.

#### Types of Interventions

The experimental group of patients with PD received VR-based therapy interventions. The types of VR interventions included nonimmersive, semi-immersive, and fully immersive approaches. Intervention media could use computers, tablets, video control platforms, mobile apps, and simulated virtual environments. There were no limits to the frequency, duration, or cycle of interventions. Control interventions consisted of routine clinical care or non-VR alternative therapies.

#### Types of Outcome Measures

The principal outcomes were global cognitive function (assessed by the Montreal Cognitive Assessment [MoCA] or the Mini-Mental State Examination [MMSE]), executive function (eg, Trail Making Test Part B, Frontal Assessment Battery, and Stroop Test), attention (eg, Trail Making Test Part A and Digit Span Forward), and memory (eg, Rey Auditory Verbal Learning Test, Verbal Memory Process Test, and Addenbrooke's Cognitive Examination–Revised [ACE-R] Memory). Secondary outcomes were depression (eg, Beck Depression Inventory, Geriatric Depression Scale, and Hamilton Depression Rating Scale) and QoL (assessed by the Parkinson's Disease Questionnaire-39).

### Exclusion Criteria

#### Types of Trials

Systematic reviews, case reports, dissertations, conference proceedings, and abstracts were explicitly excluded.

#### Types of Participants

The eligibility criteria were as follows: (1) diseases or conditions other than PD, (2) physical impairments preventing engagement in VR rehabilitation, (3) age d older than 85 years, and (4) presence of severe medical illnesses that interfere with VR training.

#### Types of Interventions

The intervention in the trial group did not include VR-based therapy, whereas the control group underwent VR-based therapy.

#### Types of Outcome Measures

Outcomes that are irrelevant, with analysis based on the same set of data.

### Study Selection and Data Extraction

Two independent reviewers conducted the study selection process in accordance with predetermined criteria and systematically abstracted data from eligible trials using a standardized Microsoft Excel template. Conflicts of opinion were settled via discussion with an experienced investigator. The information systematically abstracted from studies included the author, publication year, country, sample size, patients’ ages, details of the intervention and the control, and related outcomes.

### Risk of Bias Assessment

Two authors independently assessed the risk of bias in the included studies. The Cochrane bias assessment tool was used to examine study quality across key domains: randomization methods, allocation concealment, blinding, dropout rates, outcome reporting, and other biases [26]. Disagreements in opinion were resolved through discussion with an experienced investigator.

### Statistical Analysis

Comprehensive meta-analyses were performed using R software (version 4.4.1; R Foundation for Statistical Computing) with the meta package. For continuous variables, effect sizes were computed as standardized mean differences (SMDs), presented with 95% CIs. The weight assigned to each study in the pooled analysis was calculated using the inverse variance method. For studies reporting the median and range rather than the mean and SD, we used the methods developed by Luo et al [27] and Wan et al [28] to estimate these values [27,28]. Briefly, these methods use sample size–based formulas to derive the mean and SD from the median, range, and/or IQR, under the assumption that the underlying data are approximately normally distributed. All meta-analyses were conducted using the random-effects model. This model was chosen because we did not assume that the included studies shared a single common effect size, given the expected variations in study populations and methodologies [29]. For meta-analyses with CIs close to the line of no effect, the Knapp-Hartung adjustment was applied to provide more conservative estimates. Statistical heterogeneity among studies was assessed using the Cochran *Q* test and quantified using the *I*^2^ statistic. A *P*<.10 for the Cochran *Q* test was considered to indicate statistically significant heterogeneity, while *I*^2^ values greater than 50% were considered to represent substantial heterogeneity. We calculated PIs to quantify the real-world practical implications of heterogeneity [30]. We performed sensitivity analyses by sequentially removing each study and comparing the resulting changes in effect estimates and CIs. Because no more than 10 studies were included in each outcome, we did not create funnel plots or conduct the Egger test to assess potential small-study effects (ie, the tendency for smaller studies to show larger effect sizes than larger studies), which can be caused by publication bias, among other factors [31].

### Quality of Evidence

We applied the GRADE (Grading of Recommendations, Assessment, Development, and Evaluation) methodology to assess the certainty of evidence for each outcome measure. Given the inclusion of RCTs in our meta-analysis, we assessed the potential downgrading of evidence quality according to 5 GRADE criteria: risk of bias, inconsistency, indirectness, imprecision, and publication bias [32]. Findings were assigned to 1 of 4 evidence certainty classifications: high, moderate, low, or very low.

## Results

### Selection Process

**Figure 1.**
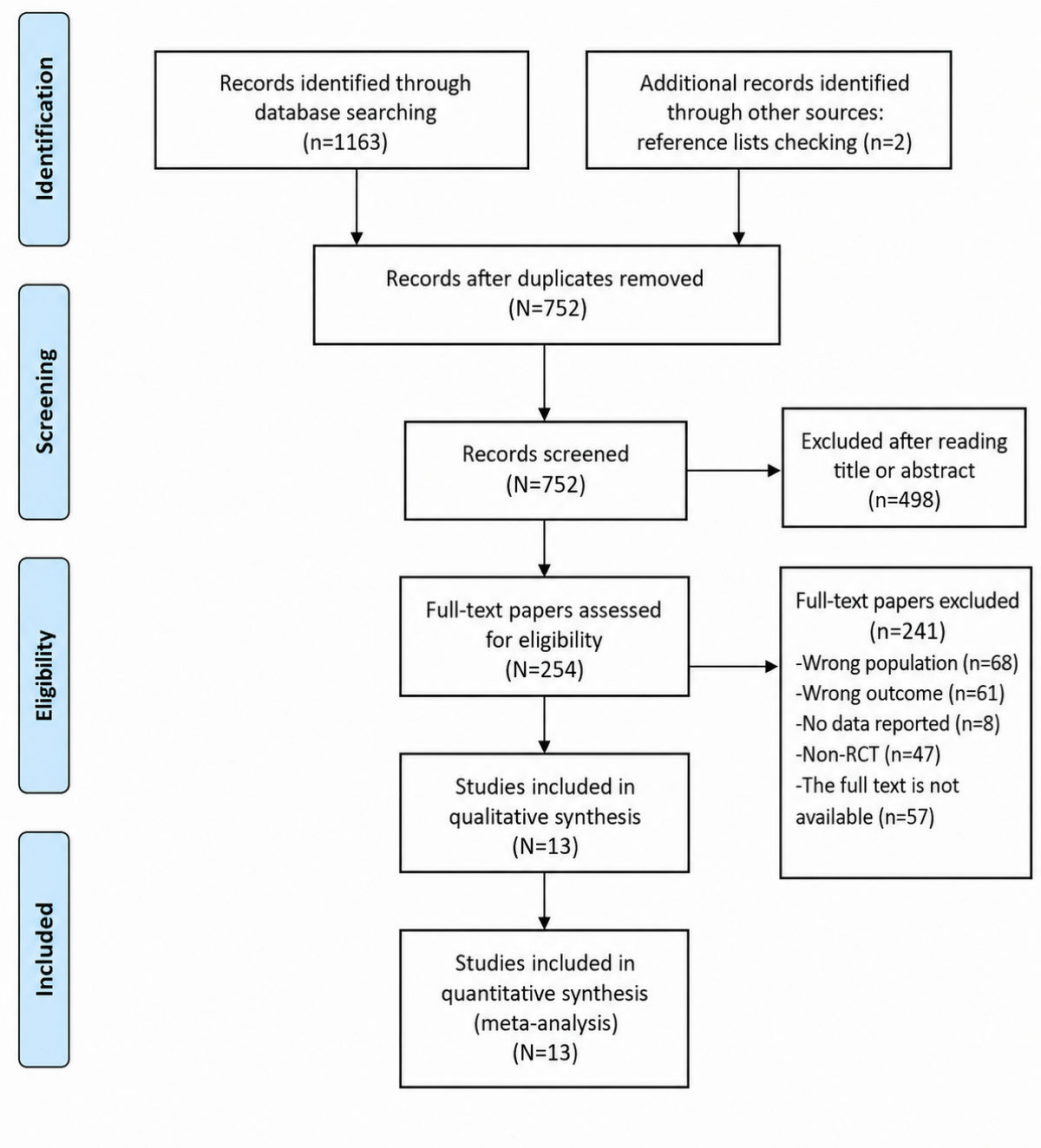
Flowchart of the study selection process. RCT: randomized controlled trial.

From the 1165 studies identified in the systematic search, after the removal of duplicate articles (n=752), 413 were left. Subsequently, 498 studies were excluded after carefully reading the titles and abstracts. Next, 241 studies were excluded after reading the full text due to the wrong population (n=68), unrelated outcomes (n=61), unreported data (n=8), non-RCTs (n=47), or unavailable full text (n=57). Ultimately, 13 RCTs were included in this meta-analysis (Figure 1).

### Study Characteristics

Details of all 13 studies are presented in Multimedia Appendix 2 [13-19,33-38]. Concerning the classification of VR-based therapies, 11 items were nonimmersive, 1 item was semi-immersive, and 1 item was immersive. The duration of intervention sessions ranged from 24 to 60 minutes. VR therapy duration differed across studies (4‐12 wk), with sessions delivered 2 to 5 times weekly. Among the 11 included studies, outcomes we focused on included global cognitive function (n=8, 62%), executive function (n=5, 38.5%), attention (n=6, 46%), memory (n=4, 30.8%), depression (5 studies, 38.5%), and QoL (n=7, 53.8%). Despite the fact that the tools used for outcome evaluation differed among trials, all had proven reliability and validity, and the data collection was executed by experienced personnel. To assess overall cognitive function, the MoCA [13,16-18,37,38] and MMSE [15,33] were used. Tools used to evaluate executive function included the Trail Making Test Part B [17,18], Frontal Assessment Battery [15,33], and Stroop Test [13]. Attention was evaluated using the Trail Making Test Part A [15,17,18,38], Dual Task Forward [13], and ACE-R Attention and Orientation [33]. Tools used to evaluate memory included the Verbal Memory Process Test [13], ACE-R Memory [33], and Rey Auditory Verbal Learning Test [15,38]. The Parkinson’s Disease Questionnaire-39 [16,18,19,33-36] was used to assess QoL. Five trials used the Geriatric Depression Scale [3,4,13,33], Hospital Anxiety and Depression Scale [19], Beck Depression Inventory [14], and Hamilton Depression Rating Scale [15] to evaluate depression levels.

### Risk of Bias Assessment

**Figure 2.**
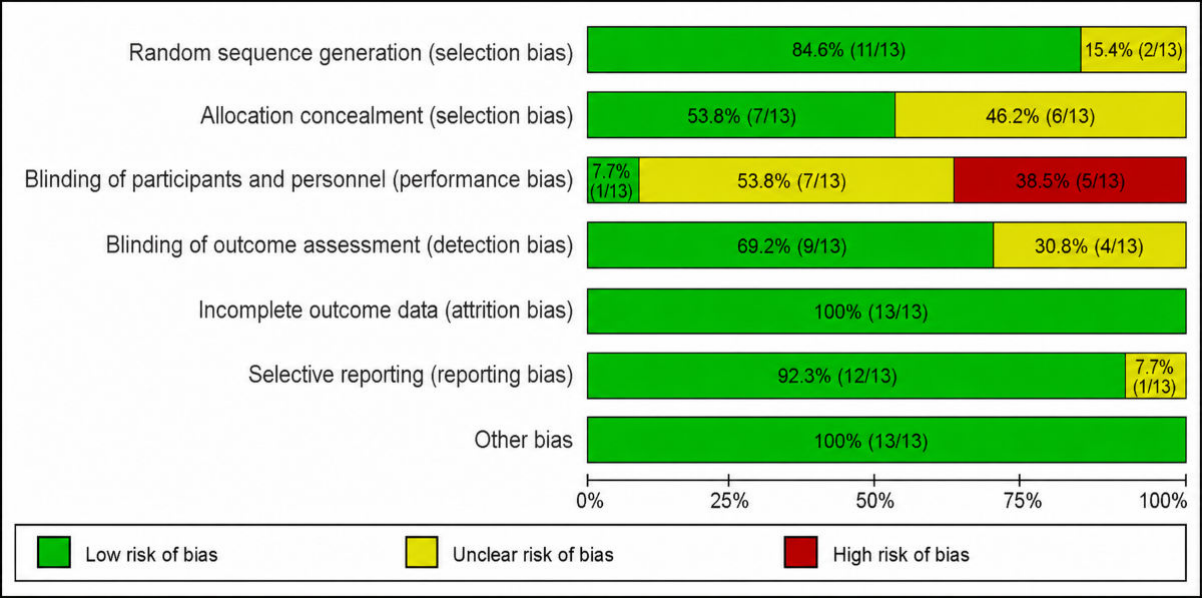
Risk of bias graph.

The recommended bias risk assessment tools from the Cochrane Handbook were rigorously applied by 2 reviewers to evaluate the risk of bias within the included studies. For instance, regarding randomization methods, 11 studies were judged as “low risk” because they implemented randomized allocation techniques, such as random number tables, stratified randomization, computer randomization programs, and coin flips. The remaining 2 studies mentioned “randomization” but did not disclose the specific randomization methods, and were evaluated as “unclear risk.” Only 3 studies achieved allocation concealment, while the remainder did not disclose whether allocation concealment was executed and were evaluated as “unclear risk.” One study that blinded patients and participants was assessed as “low risk,” while 10 of the remaining studies were rated as “high risk.” Nearly half of the trials had outcome assessors in a blinded state, and the detection bias risk in these trials was evaluated as “low risk.” Of the 13 studies, 5 had no data loss, 6 used intention-to-treat principles, and 2 used multiple regression methods to compensate for dropouts, so the risk of reporting bias was evaluated as “low risk.” Other biases were not mentioned and were evaluated as “low risk.” An overview of the methodological quality of the included papers is presented in Figures2  3.

**Figure 3.**
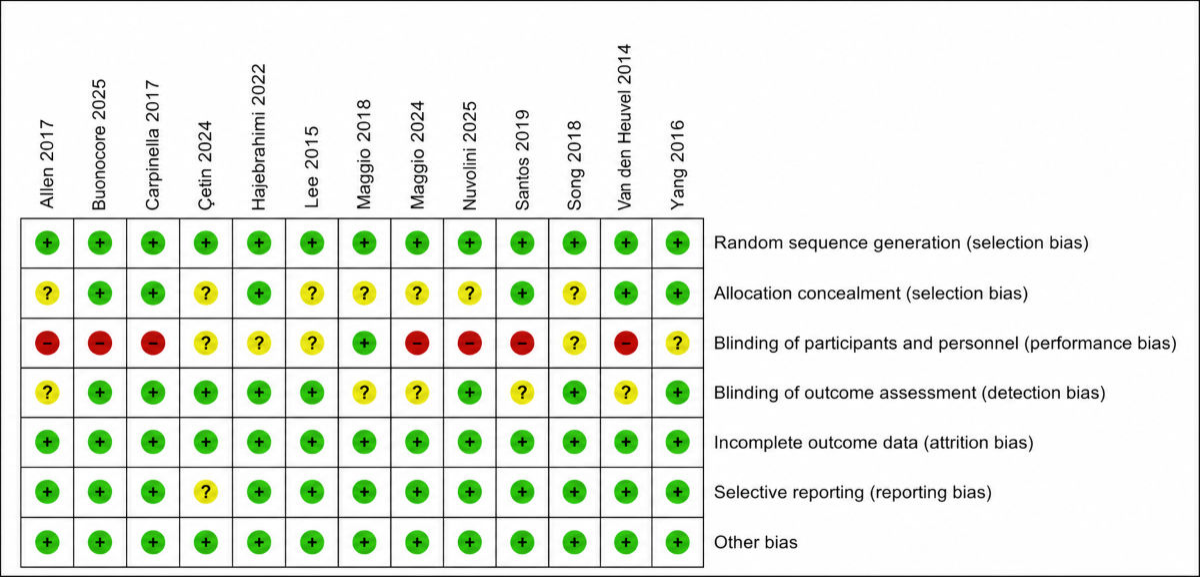
Risk of bias summary [13-19,33-38].

### Quality of Evidence

In such interventional research, complete blinding of practitioners, patients, and outcome evaluators proves particularly difficult to achieve. The included RCTs were unable to satisfy blinding criteria for either study participants or outcome assessors. As a result, the certainty of evidence was reduced by 1 level based on shortcomings in the randomized trial methodology. The overall certainty of evidence for the assessed outcomes ranged from very low to moderate quality. Comprehensive GRADE assessment results are presented in Table 1.

**Table 1.** GRADE^a^ assessment.

Certainty assessment								Patients, n		Effect, absolute (95% CI)	Certainty
Assessment	Studies, n	Study design	Risk of bias	Inconsistency	Indirectness	Imprecision	Other considerations	VR^b^-based therapy	Control		
Global cognitive function	8	Randomized trials	Not serious	Not serious	Not serious	Serious^c^	None	135	127	SMD^d^ 0.4 higher (0.11 higher to 0.70 higher)	⨁⨁⨁◯ Moderate^c^
Executive function	5	Randomized trials	Serious^e^	Not serious	Not serious	Serious^c^	None	79	77	SMD 0.06 higher (0.31 lower to 0.44 higher)	⨁⨁◯◯ Low^c^^,e^
Attention	6	Randomized trials	Serious^f^	Not serious	Not serious	Serious^c^	None	104	97	SMD 0.24 lower (0.95 lower to 0.46 higher)	⨁⨁◯◯ Low^c^,^f^
Memory	4	Randomized trials	Serious^e^	Not serious	Serious^g^	Serious^c^	None	33	33	SMD 0.48 higher (0.3 lower to 1.25 higher)	⨁◯◯◯ Very low^c^^,e,g^
Depression	5	Randomized trials	Serious^h^	Not serious	Serious^g^	Serious^c^	None	60	59	SMD 0.77 lower (1.42 lower to 0.12 lower)	⨁◯◯◯ Very low^c^^,g,h^
Quality of life	7	Randomized trials	Serious^f^	Not serious	Not serious	Serious^c^	None	98	103	SMD 0.01 higher (0.46 lower to 0.47 higher)	⨁⨁◯◯ Low^c^^, f^

a GRADE: Grading of Recommendations, Assessment, Development, and Evaluation.

b VR: virtual reality.

c The total number of events is fewer than 400, and the sample size is inadequate, leading to underpowered analyses.

d SMD: standardized mean difference.

e The lack of reported allocation concealment in some studies introduces a risk of selection bias.

f The absence of appropriate intention-to-treat analysis in some studies introduces a risk of attrition bias.

g The outcomes were measured using suboptimal methods or instruments, which may compromise the validity of the findings.

h Some studies had limitations in blinding, which raises concerns about performance bias.

### Results of the Meta-Analysis

#### Effects on Global Cognitive Function

**Figure 4.**
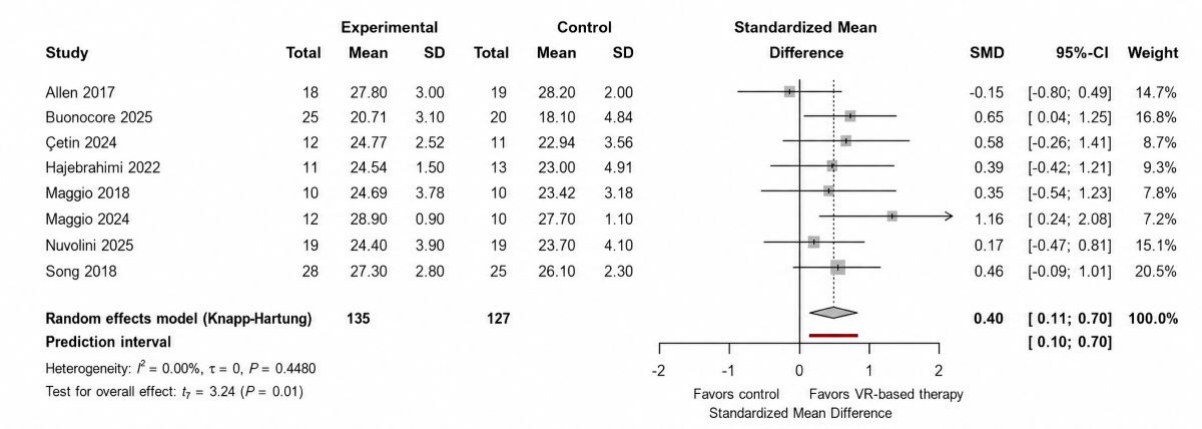
Forest plot for VR on global cognitive function. SMD: standardized mean difference; VR: virtual reality [13,15-18,33,37,38].

As shown in Figure 4, there were 8 studies [13,15-18,33,37,38] with 262 participants that examined the treatment effects of VR-mediated rehabilitation on global cognition. For global cognitive function, the pooled average effect favored VR-based therapy over control interventions (SMD=0.40, 95% CI 0.11-0.70; *I*^2^=0%; τ^2^=0). The CI indicates a statistically significant average improvement across the included studies. The PI (95% PI 0.10-0.70) also remained in the beneficial range, suggesting that similar beneficial effects may be expected across comparable future settings. However, this finding should still be interpreted cautiously because the GRADE certainty of evidence was moderate, mainly due to imprecision related to the small total sample size.

#### Effects on Executive Function

**Figure 5.**
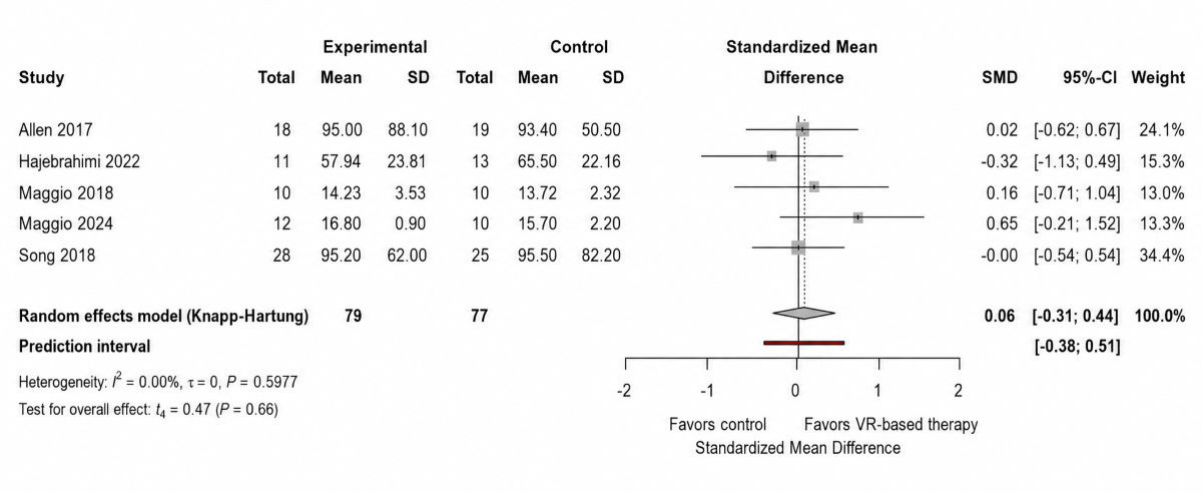
Forest plot for VR on executive function. SMD: standardized mean difference; VR: virtual reality [13,15,17,18,33].

As shown in Figure 5, there were 5 RCTs [13,15,17,18,33] with 156 participants that examined the influence of VR-based therapies on executive function. For executive function, the pooled average effect did not show a statistically significant difference between VR-based therapy and control interventions (SMD=0.06, 95% CI −0.31 to 0.44; *I*^2^=0%; τ^2^=0). Although heterogeneity was low, both the CI and PI (95% PI −0.38 to 0.51) crossed the line of no effect, indicating uncertainty in both the average effect and the likely effects across future settings. The GRADE certainty of evidence was low due to methodological concerns and imprecision.

#### Effects on Attention

**Figure 6.**
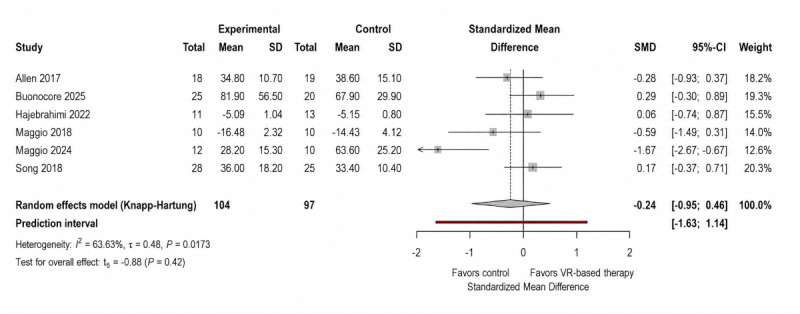
Forest plot for VR on attention. SMD: standardized mean difference; VR: virtual reality [13,15,17,18,33,38].

**Figure 7.**
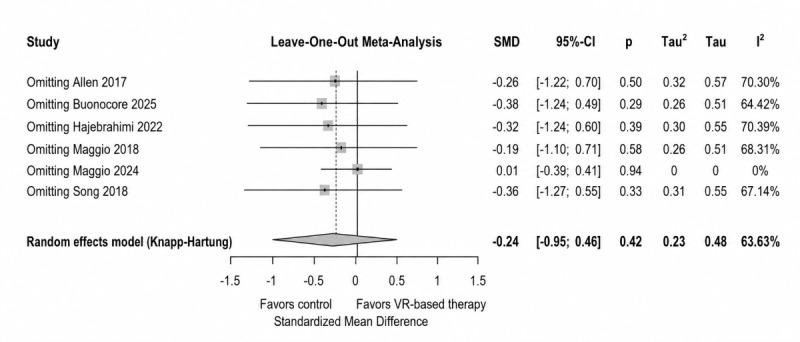
Forest plot for VR on attention after conducting sensitivity analysis [13,15,17,18,33,38]. SMD: standardized mean difference; VR: virtual reality.

There were 6 studies [13,15,17,18,33,38] with 201 patients that examined the effects of VR-mediated rehabilitation on attention, and the findings indicated no significant influence on attention compared with traditional rehabilitation (SMD=−0.24, 95% CI −0.95 to 0.46; *P*=.42; *I*^2^=64%; τ^2^=0.23; Figure 6). By excluding individual trials one by one, the *I*^2^ statistic ranged from 0% to 70%, and the 95% CI was –0.41 to 0.31. The sensitivity results showed that the sensitivity result is stable (SMD=0.01, 95% CI –0.28 to 0.31; *P*=.94; *I*^2^=0%; τ^2^=0; Figure 7). The PI (95% PI −0.95 to 0.46) also crossed 0, indicating uncertainty in the real-world effect of VR rehabilitation on attention across different populations. Given the low GRADE certainty and methodological limitations in some included studies, no firm conclusion can be drawn for this outcome.

#### Effects on Memory

**Figure 8.**
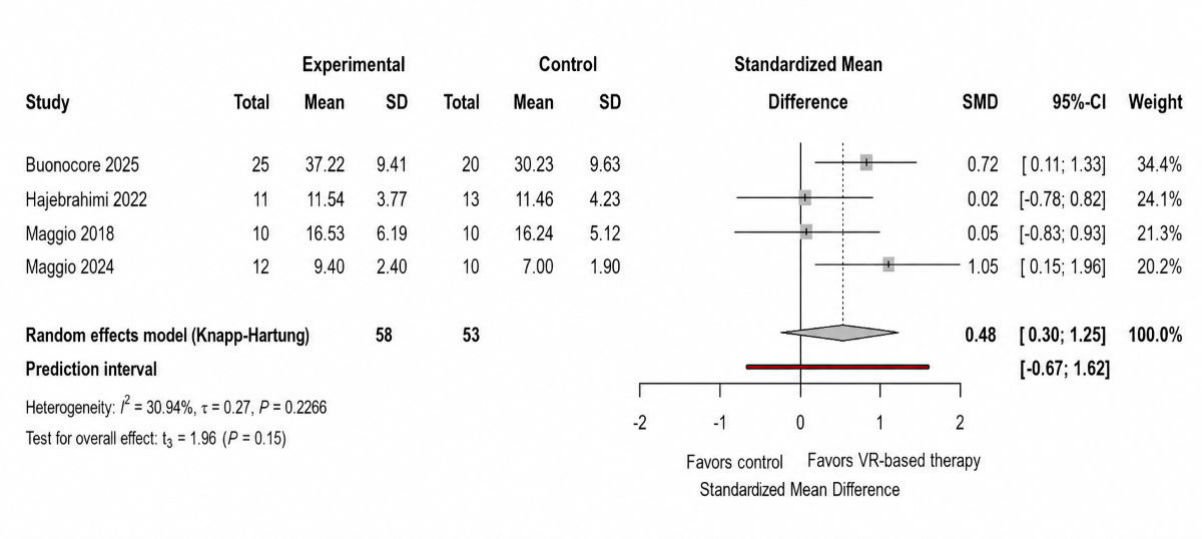
Forest plot for VR on memory. SMD: standardized mean difference; VR: virtual reality [13,15,33,38].

There were 4 studies [13,15,33,38] with 111 participants that examined the effects of VR-mediated rehabilitation on memory. For memory, the pooled average effect was not statistically significant (SMD=0.48, 95% CI −0.30 to 1.25; *I*^2^=31%; τ^2^=0.07; Figure 8). Although the point estimate favored VR-based therapy, the CI crossed the line of no effect, indicating uncertainty in the average effect. The 95% PI (95% PI −0.67 to 1.62) also crossed the line of no effect, suggesting that future studies may observe effects ranging from benefit to no benefit or possible harm. The GRADE certainty of evidence was very low, mainly due to imprecision, methodological concerns, and indirectness related to outcome measurement.

#### Effects on Depression

**Figure 9.**
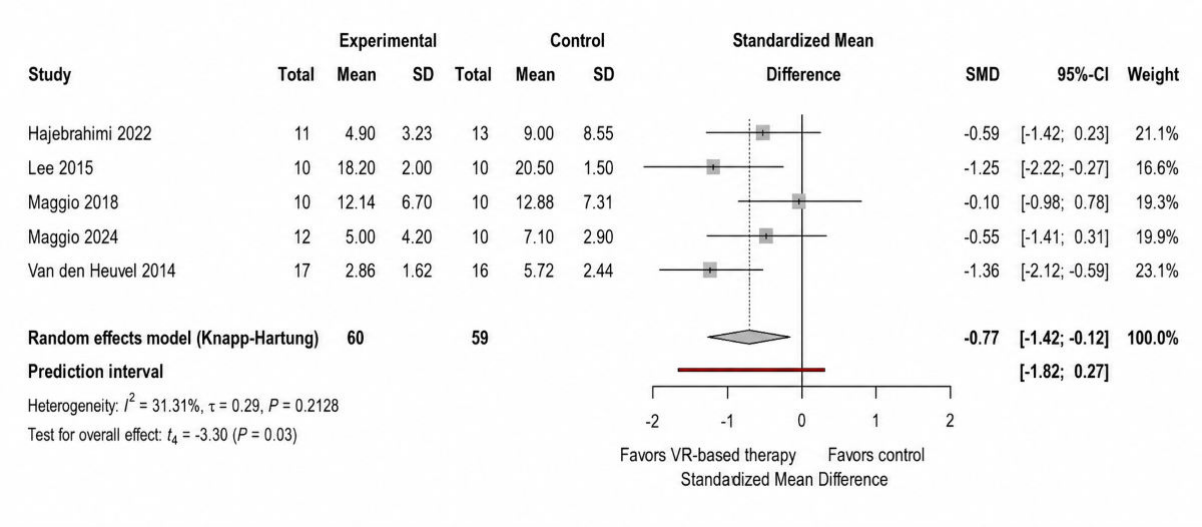
Forest plot for VR on depression. SMD: standardized mean difference; VR: virtual reality [13-15,19,33].

There were 5 RCTs [13-15,19,33] with 119 participants that investigated the effects of VR-mediated rehabilitation on depression. For depression, the pooled average effect favored VR-based therapy over control interventions (SMD=−0.77, 95% CI −1.42 to −0.12; *I*^2^=31%; τ^2^=0.10; Figure 9). The CI indicates a statistically significant average reduction in depressive symptoms. However, the PI (95% PI −1.82 to 0.27) crossed the line of no effect, indicating that the effect may not be consistently reproduced across future clinical settings. In addition, the GRADE certainty of evidence for depression was very low, with concerns related to risk of bias, imprecision, and indirectness. Therefore, this result should be interpreted as preliminary evidence of a possible average benefit.

#### Effects on QoL

**Figure 10.**
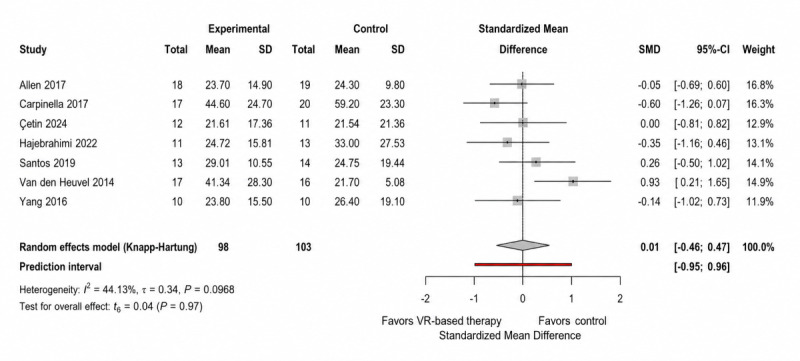
Forest plot for VR on quality of life. SMD: standardized mean difference; VR: virtual reality [13,16,18,19,34-36].

There were 7 studies [13,16,18,19,34-36] with 201 participants that examined the effects of VR-mediated rehabilitation on QoL. For QoL, no statistically significant average effect was observed between VR-based therapy and control interventions (SMD=0.01, 95% CI −0.46 to 0.47; *I*^2^=44%; τ^2^=0.11; Figure 10). Both the CI and PI (95% PI −0.95 to 0.46) crossed the line of no effect, indicating uncertainty in the average effect and substantial variability in the effects that may be observed across future settings. The GRADE certainty of evidence was low; therefore, the current evidence does not support a clear conclusion regarding the effect of VR-based therapy on QoL.

## Discussion

### Principal Findings

This systematic review and meta-analysis aimed to evaluate the effects of VR-based therapies on cognitive function and depressive symptoms in patients with PD. This systematic review and meta-analysis found that VR-based therapies were associated with statistically significant average improvements in global cognitive function and depressive symptoms in patients with PD. However, no significant improvements were observed in specific cognitive domains, such as executive function, attention, or memory. These results indicate that VR-based therapies may provide certain cognitive and psychological benefits for individuals with PD, although the current evidence remains limited.

VR-based therapy has been proven effective for cognitive rehabilitation in patients with neurocognitive disorders, demonstrating therapeutic effects on neurological recovery and performance improvement [39-41]. An increasing body of research indicates that VR-based therapy enhances the overall cognitive level of patients with PD [13,15,16,33]. Our findings also confirm that VR-based therapy positively affects the global cognitive function of patients with PD. From a neurological standpoint, the multiple sensory stimulations provided by VR promote neuroplasticity, reduce compensatory activation in areas such as the prefrontal cortex, and enhance cognitive efficiency, thus improving the overall cognitive function of patients [42]. Our results align with the meta-analysis review published by Triegaardt et al [21] and Dockx et al [12], which reported that VR therapy surpasses control interventions in enhancing the global cognition of patients with PD. However, our research outcomes differ from those of Hussain et al [43], who discovered that VR-based therapy was ineffective in improving the cognitive function of patients with PD. This discrepancy may be attributed to the inclusion of only a single VR intervention based on the Wii Fit system. Our results also diverge from those of Lei et al [20], potentially because this systematic review focused on patient gait and balance as the primary outcome indicators and included only 2 studies that targeted cognitive ability. Neither of the included studies featured VR interventions aimed at cognitive enhancement, which may have influenced the results.

Cognitive function is a complex concept, encompassing attention, executive function, memory, mental speed, and other areas of cognitive function [44]. Despite finding a positive effect on global cognitive function, our study did not identify significant improvements in the specific domains of executive function, attention, or memory. One possible explanation for this discrepancy lies in the “sum of parts” phenomenon and considerations of statistical power [45]. A significant improvement in a global composite score can occur through small, synergistic improvements across several domains that individually do not reach statistical significance in a meta-analysis. Our domain-specific analyses, especially for memory and attention, included a limited number of studies, resulting in lower statistical power to detect small-to-moderate effects. Thus, the positive trend observed in some domains might have been substantial enough, in aggregate, to drive the significant improvement in the more sensitive global cognitive score, even if each individual domain’s effect did not reach statistical significance in isolation. There are few systematic reviews of VR therapies targeting specific cognitive functions in patients with PD. A possible reason for the study results is that there are limited VR-based interventions designed to train patients with PD in specific cognitive domains. Only 1 article [33] arranges specific VR interventions for executive function, attention, and memory ability, and the results of this article show that VR has a positive impact on these 3 cognitive domains. Song et al [17] found that although objective tests of executive function did not show significant differences, patients in the intervention group subjectively reported improvement in action ability, which may indirectly reflect improvement in executive ability, especially in planning and adjusting strategies for daily activities. Although our results show that VR failed to improve executive function, attention, and memory in patients with PD, compared with traditional interventions, VR intervention can provide subjects with a wider variety of therapeutic stimulation methods. It has improved the means to monitor the effects of stimulation, enhanced subjects’ ability to interact with and respond to stimuli, and increased the standardization of treatment [46]. Therefore, research on VR for the specific cognitive functions of patients with PD is very meaningful and worth exploring in the future.

Poor mental health is a common symptom in individuals with PD, with approximately half of these individuals experiencing depression [9]. Depression affects approximately 40% of the QoL of patients with PD [47]. VR interventions have shown promise in addressing anxiety and depression, offering a safe and immersive environment where patients can learn coping strategies and manage their condition without harming those with psychological problems [48,49]. Our results indicate that VR-based therapy may improve depressive symptoms in patients with PD. One possible explanation for this enhancement is that VR training increases patients’ compliance and enthusiasm for rehabilitation. By providing multisensory stimuli and constructing a lifelike virtual environment during this engaging training process, patients are more likely to be involved and gain a sense of achievement, which is beneficial for improving their psychological state and has a positive influence on depression [49]. From a neurological perspective, VR training presents patients with interesting scenarios that stimulate the release of endorphins and dopamine, thereby generating positive emotions and enhancing their emotional state [15]. Although the pooled analysis supports the overall efficacy of VR rehabilitation for depression, the PI crossed the line of no effect, and the GRADE certainty was very low. This indicates that the effect on depressive symptoms may vary across populations, intervention protocols, and clinical contexts. Therefore, the evidence for depression should be considered preliminary. Despite improvements in depression symptoms with VR intervention, our results indicate that the QoL of patients with PD does not improve. This is consistent with the findings of Navarro-Lozano et al [50], who suggested that the efficacy of VR-based therapy in enhancing the QoL for patients with PD is inconclusive. The QoL of patients with PD is influenced not only by psychological health but also by the disease itself, social support, and environmental factors [51]. VR intervention does not have a fundamental impact on the disease process or environmental factors, and since QoL is affected by multiple factors, merely improving mental health does not necessarily benefit the overall QoL of patients.

### Limitations

Our review had some potential limitations. First, the number of trials encompassed in the meta-analysis was restricted. Second, studies varied in the types of VR systems used, the media presented, and how outcomes were assessed. Research into the optimal method of VR intervention is complex and difficult. Third, the interpretation of results is constrained by the composite nature of global cognitive scales (eg, MoCA, MMSE) versus the limited domain-specific evidence available. While the observed global improvements suggest a generalized positive effect of VR, our domain-specific meta-analyses were necessarily limited to attention, executive function, and memory due to insufficient data reported for other domains (eg, language, visuospatial ability) in the literature. Fourth, and importantly, we were unable to perform meaningful subgroup analyses to explore the potential influence of patient characteristics (eg, disease severity, baseline cognitive status) or intervention features (eg, level of immersion) on the outcomes. This was primarily due to the lack of stratified data reporting in the original studies and the insufficient number of studies in potential subgroups (eg, only 2 studies used semi-immersive VR systems). Finally, in our review, we only considered the postintervention effect and did not focus on the subsequent effects on patients. Further research is required to ascertain whether the effects observed in the present review are sustained over time. If not, research is needed to establish how often and how long VR therapy should be administered to maintain its benefits.

### Conclusions

This systematic review and meta-analysis suggest that VR-based therapies may show potential average benefits for global cognitive function and depressive symptoms, but evidence remains uncertain for executive function, attention, memory, and QoL. These results should be interpreted cautiously because of methodological limitations, low or very low certainty for several outcomes, and variability across settings, particularly for depression. Unlike previous reviews that mainly focused on motor outcomes, this review separately examined global cognitive function, specific cognitive domains, depressive symptoms, and QoL, while incorporating recent RCTs. Therefore, the current evidence suggests that VR-based therapies are a promising adjunctive rehabilitation approach, but it is insufficient to draw definitive conclusions about efficacy. The confirmation of these effects is necessitated by larger RCTs. The achievement of high-quality studies will eventually further the understanding of the ideal cognitive and mental rehabilitation approaches for patients with PD.

## Supplementary material

10.2196/77875 Multimedia Appendix 1 Search strategy.

10.2196/77875 Multimedia Appendix 2 Characteristics of the included papers.

10.2196/77875 Checklist 1 PRISMA checklist.

## Abbreviations

ACE-R: Addenbrooke's Cognitive Examination–Revised
GRADE: Grading of Recommendations, Assessment, Development, and Evaluation
MMSE: Mini-Mental State Examination
MoCA: Montreal Cognitive Assessment
PD: Parkinson disease
PI: prediction interval
PRISMA: Preferred Reporting Items for Systematic Reviews and Meta-Analyses
QoL: quality of life
RCT: randomized controlled trial
SMD: standardized mean difference
VR: virtual reality
