# Supplementary material for: Impact of Virtual Reality–Based Therapies on Cognition and Depression in Patients With Parkinson Disease: Systematic Review and Meta-Analysis of Randomized Controlled Trials
Source: JMIR Serious Games. 2026 Jun 30;14:e77875. doi: 10.2196/77875 (PMC13318082; doi:10.2196/77875)
Supplement: Multimedia Appendix 1 [file games-v14-e77875-s001.docx]

**Multimedia Appendix 1.** Details of the search.

**PUBMED**

| **#** | **Search terms** | **Results** |
| --- | --- | --- |
| 1 | ("Parkinson Disease"[MeSH Terms] OR Parkinson*[tiab] OR "Parkinson disease"[tiab] OR "Parkinson's disease"[tiab] OR "Parkinsonism"[tiab] OR "parkinsonian"[tiab] OR "PD"[tiab]) | 355,827 |
| 2 | ("Virtual Reality"[MeSH Terms] OR "virtual reality"[tiab] OR "virtual realities"[tiab] OR VR[tiab] OR "virtual environment*"[tiab] OR "virtual training"[tiab] OR "virtual rehabilitation"[tiab] OR "VR-based"[tiab] OR "video game*"[tiab] OR videogame*[tiab] OR "computer game*"[tiab] OR exergam*[tiab] OR "interactive game*"[tiab] OR "serious game*"[tiab] OR "augmented reality"[tiab] OR "mixed reality"[tiab] OR "immersive technolog*"[tiab] OR "Nintendo Wii"[tiab] OR Wii[tiab] OR Kinect[tiab]) | 58,533 |
| 3 | ("Cognition"[MeSH Terms] OR "Cognitive Dysfunction"[MeSH Terms] OR "Neuropsychological Tests"[MeSH Terms] OR "Depression"[MeSH Terms] OR "Depressive Disorder"[MeSH Terms] OR "Mood Disorders"[MeSH Terms] OR cognit*[tiab] OR cognition[tiab] OR "cognitive function*"[tiab] OR "cognitive impairment"[tiab] OR "cognitive dysfunction"[tiab] OR "executive function*"[tiab] OR attention[tiab] OR memory[tiab] OR "global cognition"[tiab] OR neuropsycholog*[tiab] OR MoCA[tiab] OR "Montreal Cognitive Assessment"[tiab] OR MMSE[tiab] OR "Mini-Mental State Examination"[tiab] OR depress*[tiab] OR mood[tiab] OR "mood disorder*"[tiab] OR affective[tiab] OR anxiety[tiab] OR "emotional well-being"[tiab] OR HADS[tiab] OR BDI[tiab] OR "Beck Depression Inventory"[tiab] OR HAM-D[tiab] OR "Hamilton Depression"[tiab] OR "Geriatric Depression Scale"[tiab] OR GDS[tiab]) | 2,508,914 |
| 4 | (randomized controlled trial[pt] OR controlled clinical trial[pt] OR randomized[tiab] OR randomised[tiab] OR randomly[tiab] OR trial[tiab] OR placebo[tiab] OR groups[tiab]) NOT (animals[mh] NOT humans[mh]) | 3,929,961 |
| 5 | #1 AND #2 AND #3 AND #4 | 163 |

**Web of Science**

| **#** | **Search terms** | **Results** |
| --- | --- | --- |
| 1 | TS=(Parkinson* OR "Parkinson disease" OR "Parkinson's disease" OR Parkinsonism OR parkinsonian OR PD) | 636,931 |
| 2 | TS=("virtual reality" OR "virtual realities" OR VR OR "virtual environment*" OR "virtual training" OR "virtual rehabilitation" OR "VR-based" OR "video game*" OR videogame* OR "computer game*" OR exergam* OR "interactive game*" OR "serious game*" OR "augmented reality" OR "mixed reality" OR "immersive technolog*" OR "Nintendo Wii" OR Wii OR Kinect) | 236,335 |
| 3 | TS=(cognit* OR cognition OR "cognitive function*" OR "cognitive impairment" OR "cognitive dysfunction" OR "executive function*" OR attention OR memory OR "global cognition" OR neuropsycholog* OR MoCA OR "Montreal Cognitive Assessment" OR MMSE OR "Mini-Mental State Examination" OR depress* OR mood OR "mood disorder*" OR affective OR anxiety OR "emotional well-being" OR HADS OR BDI OR "Beck Depression Inventory" OR HAM-D OR "Hamilton Depression" OR "Geriatric Depression Scale" OR GDS) | 5,069,236 |
| 4 | TS=(random* OR randomised OR randomized OR randomly OR trial OR placebo OR groups OR crossover OR "cross over" OR "controlled trial" OR "clinical trial") | 11,329,548 |
| 5 | #1 AND #2 AND #3 AND #4 | 329 |

**Cochrane Library**

| **#** | **Search terms** | **Results** |
| --- | --- | --- |
| 1 | MeSH descriptor: [Virtual Reality Exposure Therapy] explode all trees | 466 |
| 2 | ('virtual reality immersion therapy' OR 'vr exposure therapy' OR 'vr immersion therapy' OR 'virtual reality therapy' OR 'reality therapies, virtual' OR 'reality therapy, virtual' OR 'therapies, virtual reality' OR 'therapy, virtual reality' 'virtual reality therapies' OR 'virtual reality' OR 'video gam*' OR 'computer gam*' OR 'gaming consol*' OR 'interactive gam*' OR 'xbox' OR 'plantation' OR 'nintendo' OR 'serious game*'):ab,ti, | 14627 |
| 3 | #1OR#2 | 14657 |
| 4 | MeSH descriptor: [Parkinson Disease] explode all trees | 6603 |
| 5 | ('idiopathic parkinsonism' or 'Lewy bodies of Parkinson disease' or 'Lewy bodies of Parkinsons disease' or 'Lewy body Parkinson disease' or 'Lewy body Parkinsons disease' or 'paralysis agitans' or 'Parkinson dementia complex' or 'Parkinsons disease' or 'primary parkinsonism' or 'Parkinson disease'):ab,ti | 14051 |
| 6 | #4OR#5 | 14675 |
| 7 | MeSH descriptor: [Cognition] explode all trees | 17481 |
| 8 | ('cognit*' or 'cognitive function*' or 'cognitive impairment' or 'cognitive dysfunction' or 'executive function*' or 'attention' or 'memory' or 'global cognition' or neuropsycholog' or 'MoCA' or 'Montreal Cognitive Assessment'):ab,ti | 162985 |
| 9 | MeSH descriptor: [Depression] explode all trees | 20440 |
| 10 | ('depress*' or 'mood' or 'mood disorder*' or 'affective' or 'emotional well-being' or 'HADS' or 'BDI' or 'Beck Depression Inventory' or HAM-D' or 'Hamilton Depression' or 'Geriatric Depression Scale' or 'GDS'):ab,ti | 148239 |
| 11 | #7 OR #8 OR #9 OR #10 | 274669 |
| 12 | #3 AND #6 AND #11 | 172 |

**Embase**

| **#** | **Search terms** | **Results** |
| --- | --- | --- |
| 1 | 'parkinson disease'/exp OR 'parkinson disease' OR 'idiopathic parkinsonism':ab,ti OR 'lewy bodies of parkinson disease':ab,ti OR 'lewy bodies of parkinsons disease':ab,ti OR 'lewy body parkinson disease':ab,ti OR 'lewy body parkinsons disease':ab,ti OR 'paralysis agitans':ab,ti OR 'parkinson dementia complex':ab,ti OR 'parkinsons disease':ab,ti OR 'primary parkinsonism':ab,ti OR 'parkinson disease':ab,ti | 250166 |
| 2 | 'virtual reality'/exp OR 'augmented reality'/exp OR 'mixed reality'/exp OR 'video game'/exp OR 'computer game'/exp OR 'virtual reality':ti,ab,kw OR 'virtual realities':ti,ab,kw OR vr:ti,ab,kw OR 'virtual environment*':ti,ab,kw OR 'virtual training':ti,ab,kw OR 'virtual rehabilitation':ti,ab,kw OR 'vr-based':ti,ab,kw OR 'video game*':ti,ab,kw OR videogame*:ti,ab,kw OR 'computer game*':ti,ab,kw OR exergam*:ti,ab,kw OR 'interactive game*':ti,ab,kw OR 'serious game*':ti,ab,kw OR 'augmented reality':ti,ab,kw OR 'mixed reality':ti,ab,kw OR 'immersive technolog*':ti,ab,kw OR 'nintendo wii':ti,ab,kw OR wii:ti,ab,kw OR kinect:ti,ab,kw | 94258 |
| 3 | 'cognition'/exp OR 'cognitive defect'/exp OR 'cognitive impairment'/exp OR 'neuropsychological test'/exp OR 'depression'/exp OR 'depressive disorder'/exp OR 'mood disorder'/exp OR cognit*:ti,ab,kw OR cognition:ti,ab,kw OR 'cognitive function*':ti,ab,kw OR 'cognitive impairment':ti,ab,kw OR 'cognitive dysfunction':ti,ab,kw OR 'executive function*':ti,ab,kw OR attention:ti,ab,kw OR memory:ti,ab,kw OR 'global cognition':ti,ab,kw OR neuropsycholog*:ti,ab,kw OR moca:ti,ab,kw OR 'montreal cognitive assessment':ti,ab,kw OR mmse:ti,ab,kw OR 'mini mental state examination':ti,ab,kw OR depress*:ti,ab,kw OR mood:ti,ab,kw OR 'mood disorder*':ti,ab,kw OR affective:ti,ab,kw OR anxiety:ti,ab,kw OR 'emotional well-being':ti,ab,kw OR hads:ti,ab,kw OR bdi:ti,ab,kw OR 'beck depression inventory':ti,ab,kw OR 'hamilton depression':ti,ab,kw OR 'geriatric depression scale':ti,ab,kw OR gds:ti,ab,kw | 6294169 |
| 4 | 'randomized controlled trial'/exp OR 'controlled clinical trial'/de OR random*:ti,ab,kw OR randomised:ti,ab,kw OR randomized:ti,ab,kw OR randomly:ti,ab,kw OR placebo:ti,ab,kw OR trial:ti,ab,kw OR groups:ti,ab,kw OR crossover:ti,ab,kw OR 'cross over':ti,ab,kw OR 'double blind procedure'/de OR 'single blind procedure'/de | 7216206 |
| 5 | #1 AND #2 AND #3 AND #4 | 499 |
