# Supplementary material for: Impact of Virtual Reality–Based Therapies on Cognition and Depression in Patients With Parkinson Disease: Systematic Review and Meta-Analysis of Randomized Controlled Trials
Source: JMIR Serious Games. 2026 Jun 30;14:e77875. doi: 10.2196/77875 (PMC13318082; doi:10.2196/77875)
Supplement: Multimedia Appendix 2 [file games-v14-e77875-s002.docx]

**Multimedia Appendix 2 Summarizes the characteristics of the included studies in the meta-analysis.**

| **Study/Year** | **Mean age** | **Number** | **Intervention measures** | **Frequency** | **Country** | **Measure** | **Effect size and CI** |
| --- | --- | --- | --- | --- | --- | --- | --- |
| **Global cognitive function [13,15-18,33,37,38]** | | | | | | | |
| Allen/2017 | I: 67.5±7.3  C: 68.4±8.5 | I:18  C:19 | **I**^a^**:** Non - immersive VR^b^ Intervention,  Arm and Hand Coordination Training Based on Unity Software  **C**^c^**:** Conventional arm and hand training | 24 - 36 min/time, 3 times/week, 12 weeks | Australia | MoCA^d^ | −0.15 [−0.80, 0.49] |
| Hajebrahimi/ 2022 | I: 66.3±8.04  C: 65.5±9.93 | I:11  C:13 | **I:** Non - immersive VR Intervention, Balance and Gait Training Based on Wii System  **C:** Conventional balance training | 60 min/time, 3 times/week, 4 weeks | Turkey | MoCA | 0.39 [−0.42, 1.21] |
| Maggio/2018 | I: 69.9±6.3  C: 68.9±10.05 | I:10  C:10 | **I:** Semi - immersive VR Intervention, Cognitive Training Based on BTS Nirvana System  **C:** Routine cognitive training | 60 min/time, 3 times/week, 8 weeks | Australia | MMSE^e^ | 0.35 [−0.54, 1.23] |
| Maggio/2024 | I: 59.7±9.7  C: 66.8±6.5 | I:12  C:10 | **I:** Non - immersive VR Intervention, Cognitive Training Based on VR Applications  **C:** Routine cognitive training | 30 min/time, 3 times/week, 6 weeks | Australia | MMSE | 1.16 [0.24, 2.08] |
| Song/2018 | I: 68±7  C: 65±7 | I:28  C:25 | **I:** Non - immersive VR Intervention, Gait and Balance Training Intervention Based on the Video Game Stepmania  **C:** Conventional balance training | 15 min/time, 3 times/week, 12 weeks | Australia | MoCA | 0.46 [−0.09, 1.01] |
| Çetin/2024 | I: 54~73  C: 51~84 | I:12  C:11 | **I:** Non - immersive VR Intervention: Motor and Cognitive Intervention Based on the USE - IT Intelligent System  **C:** Routine motor and cognitive intervention | 60 min/time, 3 times/week, 8 weeks | Turkey | MoCA | 0.58 [−0.26, 1.41] |
| Buonocore 2025 | I: 69.6±6.1  C: 72.7±7.8 | I:25  C:20 | **I:** Non - immersive VR Intervention: Motor and Cognitive Intervention Based on VRRS - HomeKit  **C:** Traditional face-to-face cognitive stimulation training | 45 min/time, 5 times/week, 4 weeks | Italy | MoCA | 0.65 [0.04, 1.25] |
| Nuvolini 2025 | I: 62.7±6.8  C: 69.2±7.8 | I:19  C:19 | **I:** Non - immersive VR Intervention: Motor and Cognitive Intervention Based on Kinect Adventures  **C:** Traditional motor and cognitive intervention | 60 min/time, 2 times/week, 7 weeks | Brazil | MoCA | 0.17 [−0.47, 0.81] |
| **Executive function [13,15,17,18,33]** | | | | | | | |
| Allen/2017 | I: 67.5±7.3  C: 68.4±8.5 | I:18  C:19 | **I:** Non - immersive VR Intervention,  Arm and Hand Coordination Training Based on Unity Software  **C:** Conventional arm and hand training | 24 - 36 min/time, 3 times/week, 12 weeks | Australia | TMT-B^f^ | 0.02 [−0.62, 0.67] |
| Hajebrahimi/ 2022 | I: 66.3±8.04  C: 65.5±9.93 | I:11  C:13 | **I:** Non - immersive VR Intervention, Balance and Gait Training Based on Wii System  **C:** Conventional balance training | 60 min/time, 3 times/week, 4 weeks | Turkey | Stroop TD | −0.32 [−1.13, 0.49] |
| Maggio/2018 | I: 69.9±6.3  C: 68.9±10.05 | I:10  C:10 | **I:** Semi - immersive VR Intervention, Cognitive Training Based on BTS Nirvana System  **C:** Routine cognitive training | 60 min/time, 3 times/week, 8 weeks | Australia | FAB^g^ | 0.16 [−0.71, 1.04] |
| Maggio/2024 | I: 59.7±9.7  C: 66.8±6.5 | I:12  C:10 | **I:** Non - immersive VR Intervention, Cognitive Training Based on VR Applications  **C:** Routine cognitive training | 30 min/time, 3 times/week, 6 weeks | Australia | FAB | 0.65 [−0.21, 1.52] |
| Song/2018 | I: 68±7  C: 65±7 | I:28  C:25 | **I:** Non - immersive VR Intervention, Gait and Balance Training Intervention Based on the Video Game Stepmania  **C:** Conventional balance training | 15 min/time, 3 times/week, 12 weeks | Australia | TMT-B | −0.00 [−0.54, 0.54] |
| **Attention [13,15,17,18,33,38]** | | | | | | | |
| Allen/2017 | I: 67.5±7.3  C: 68.4±8.5 | I:18  C:19 | **I:** Non - immersive VR Intervention,  Arm and Hand Coordination Training Based on Unity Software  **C:** Conventional arm and hand training | 24 - 36 min/time, 3 times/week, 12 weeks | Australia | TMT-A^h^ | −0.28 [−0.93, 0.37] |
| Buonocore 2025 | I: 69.6±6.1  C: 72.7±7.8 | I:25  C:20 | **I:** Non - immersive VR Intervention: Motor and Cognitive Intervention Based on VRRS - HomeKit  **C:**Traditional face-to-face cognitive stimulation training | 45 min/time, 5 times/week, 4 weeks | Italy | TMT-A | 0.29 [−0.30, 0.89] |
| Hajebrahimi/ 2022 | I: 66.3±8.04  C: 65.5±9.93 | I:11  C:13 | **I:** Non - immersive VR Intervention, Balance and Gait Training Based on Wii System  **C:** Conventional balance training | 60 min/time, 3 times/week, 4 weeks | Turkey | DS forward^i^ | 0.06 [−0.74, 0.87] |
| Maggio/2018 | I: 69.9±6.3  C: 68.9±10.05 | I:10  C:10 | **I:** Semi - immersive VR Intervention, Cognitive Training Based on BTS Nirvana System  **C:** Routine cognitive training | 60 min/time, 3 times/week, 8 weeks | Australia | ACE-R AO^j^ | −0.59 [−1.49, 0.31] |
| Maggio/2024 | I: 59.7±9.7  C: 66.8±6.5 | I:12  C:10 | **I:** Non - immersive VR Intervention, Cognitive Training Based on VR Applications  **C:** Routine cognitive training | 30 min/time, 3 times/week, 6 weeks | Australia | TMT-A | −1.675 [−2.67, −0.67] |
| Song/2018 | I: 68±7  C: 65±7 | I:28  C:25 | **I:** Non - immersive VR Intervention, Gait and Balance Training Intervention Based on the Video Game Stepmania  **C:** Conventional balance training | 15 min/time, 3 times/week, 12 weeks | Australia | TMT-A | 0.17 [−0.37, 0.71] |
| **Memory [13,15,33,38]** | | | | | | | |
| Hajebrahimi/ 2022 | I: 66.36±8.04  C: 65.53±9.93 | I:11  C:13 | **I:** Non - immersive VR Intervention, Balance and Gait Training Based on Wii System  **C:** Conventional balance training | 60 min/time, 3 times/week, 4 weeks | Turkey | VMPT^k^ | 0.02 [−0.78, 0.82] |
| Maggio/2018 | I: 69.9±6.3  C: 68.9±10.05 | I:10  C:10 | **I:** Semi - immersive VR Intervention, Cognitive Training Based on BTS Nirvana System  **C:** Routine cognitive training | 60 min/time, 3 times/week, 8 weeks | Australia | ACE-R M^l^ | 0.05 [−0.83, 0.93] |
| Buonocore 2025 | I: 69.6±6.1  C: 72.7±7.8 | I:25  C:20 | **I:** Non - immersive VR Intervention: Motor and Cognitive Intervention Based on VRRS - HomeKit  **C:** Traditional face-to-face cognitive stimulation training | 45 min/time, 5 times/week, 4 weeks | Italy | RAVLT^m^ | 0.72 [0.11, 1.33] |
| Maggio/2024 | I: 59.7±9.7  C: 66.8±6.5 | I:12  C:10 | **I:** Non - immersive VR Intervention, Cognitive Training Based on VR Applications  **C:** Routine cognitive training | 30 min/time, 3 times/week, 6 weeks | Australia | RAVLT | 1.05 [0.15, 1.96] |
| **Depression [13-15,19,33]** | | | | | | | |
| Hajebrahimi/ 2022 | I: 66.36±8.04  C: 65.53±9.93 | I:11  C:13 | **I:** Non - immersive VR Intervention, Balance and Gait Training Based on Wii System  **C:** Conventional balance training | 60 min/time, 3 times/week, 4 weeks | Turkey | GDS^n^ | −0.59 [−1.42, 0.23] |
| Lee/2015 | I: 53.3±11.8  C: 54.6±13.4 | I:16  C:16 | **I:** Non - immersive VR Intervention, Balance and Motor Training Based on the K - Pop Game of the Wii System  **C:** Functional Electrical Stimulation | 45 min/time, 5 times/week, 6 weeks | Korea | BDI^o^ | −1.30 [−2.29, −0.32] |
| Maggio/2018 | I: 69.9±6.3  C: 68.9±10.05 | I:10  C:10 | **I:** Semi - immersive VR Intervention, Cognitive Training Based on BTS Nirvana System  **C:** Routine cognitive training | 60 min/time, 3 times/week, 8 weeks | Australia | BDI | −0.10 [−0.98, 0.78] |
| Maggio/2024 | I: 59.7±9.7  C: 66.8±6.5 | I:12  C:10 | **I:** Non - immersive VR Intervention, Cognitive Training Based on VR Applications  **C:** Routine cognitive training | 30 min/time, 3 times/week, 6 weeks | Australia | HAM-D^p^ | −0.55 [−1.41, 0.31] |
| Van den Heuvel/ 2014 | I: 66.3±6.39  C: 68.8±9.68 | I:17  C:16 | **I:** Non - immersive virtual reality, Balance training based on enhanced visual feedback  **C:** Conventional balance training | 60 min/time, 2 times/week, 5 weeks | Netherlands | HADS-D^q^ | −1.36 [−2.12, −0.59] |
| **Quality of life [13,16,18,19,34-36]** | | | | | | | |
| Allen/2017 | I: 67.5±7.3  C: 68.4±8.5 | I:18  C:19 | **I：**Non - immersive VR Intervention,  Arm and Hand Coordination Training Based on Unity Software  **C:** Conventional arm and hand training | 24 - 36 min/time, 3 times/week, 12 weeks | Australia | PDQ-39^r^ | −0.05 [−0.69,0.60] |
| Carpinella/2017 | I: 73.0±7.1  C: 75.6±8.2 | I:17  C:20 | **I:** Fully Immersive VR Intervention, Balance and Gait Training Based on the Biofeedback System (Gamepad) of Wearable Sensors  **C:** Traditional physical training | 45 min/time, 3 times/week, 6 weeks | Australia | PDQ-39 | −0.60 [−1.26, 0.07] |
| Hajebrahimi/ 2022 | I: 66.36±8.04  C: 65.53±9.93 | I:11  C:13 | **I:** Non - immersive VR Intervention, Balance and Gait Training Based on Wii System  **C:** Conventional balance training | 60 min/time, 3 times/week, 4 weeks | Turkey | PDQ-39 | −0.35 [−1.16, 0.46] |
| Santos/2019 | I: 61.7±7.3  C: 64.5±9.8 | I:13  C:14 | **I:** Non - immersive VR Intervention, Motor Training Based on the Wii System  **C:** Conventional exercise training | 40 min/time, 2 times/week, 8 weeks | Brazil | PDQ-39 | 0.26 [−0.50, 1.02] |
| Van den Heuvel/ 2014 | I: 66.3±6.39  C: 68.8±9.68 | I:17  C:16 | **I:** Non - immersive virtual reality, Balance training based on enhanced visual feedback  **C:** Routine balance training | 60 min/time, 2 times/week, 5 weeks | Netherlands | PDQ-39 | 0.93 [0.21, 1.65] |
| Yang/2016 | I: 72.5±8.4  C: 75.4±6.3 | I:10  C:10 | **I:** Non - immersive VR Intervention, Balance Training Based on a Customized Touchscreen and Balance Board System  **C:** Conventional balance training | 50 min/time, 2 times/week, 6 weeks | China | PDQ-39 | −0.14 [−1.02, 0.73] |
| Çetin/2024 | I: 65-85  C: 65-85 | I:12  C:11 | **I:** Non - immersive VR Intervention: Motor and Cognitive Intervention Based on the USE - IT Intelligent System  **C:** Routine motor and cognitive intervention | 60 min/time, 3 times/week, 8 weeks | Turkey | PDQ-39 | 0.00 [−0.81, 0.82] |

^a^I: Intervention Group.

^b^VR: Virtual Reality.

^c^C: Control Group.

^d^MoCA: Montreal Cognitive Assessment Scale.

^e^MMSE: Mini-Mental State Examination.

^f^TMT-B: Trail Making Test Part B.

^g^FAB: Frontal Assessment Battery.

^h^TMT-A: Trail Making Test Part A.

^i^DS forward: Dual Task forward.

^j^ACE-R AO: ACE-R Attention and Orientation.

^k^VMPT: Verbal Memory Process Test.

^l^ACE-R M: ACE-R Memory.

^m^RAVLT: Rey Auditory Verbal Learning Test.

^n^GDS: Geriatric Depression Scale.

^o^BDI: Beck Depression Inventory.

^p^HAM-D: Hamilton Depression Rating Scale.

^q^HADS-D: Hospital Anxiety and Depression Scale.

^r^PDQ-39: Parkinson's Disease Questionnaire-39.
